# High Ki-67 index in fine needle aspiration cytology of follicular thyroid tumors is associated with increased risk of carcinoma

**DOI:** 10.1007/s12020-018-1627-z

**Published:** 2018-05-23

**Authors:** Ninni Mu, C. Christofer Juhlin, Edneia Tani, Anastasios Sofiadis, Eva Reihnér, Jan Zedenius, Catharina Larsson, Inga-Lena Nilsson

**Affiliations:** 1 Department of Oncology-Pathology, Karolinska Institutet, Stockholm, Sweden; 2 Cancer Center Karolinska, Karolinska University Hospital, Stockholm, Sweden; 3 Department of Pathology and Cytology, Karolinska University Hospital, Stockholm, Sweden; 4 Department of Oncology, Karolinska University Hospital, Stockholm, Sweden; 5 Department of Molecular Medicine and Surgery, Karolinska Institutet, Stockholm, Sweden; 6 Department of Breast, Endocrine Tumours and Sarcoma, Karolinska University Hospital, Stockholm, Sweden

**Keywords:** Follicular thyroid tumors, Ki-67, Proliferation, Cytology

## Abstract

**Purpose:**

Preoperative distinction of follicular thyroid carcinoma (FTC) from follicular thyroid adenoma (FTA) is a diagnostic challenge. Our aim was to investigate whether the Ki-67 proliferation index in fine needle aspiration material can contribute to the diagnosis of FTC.

**Methods:**

We analyzed retrospectively cytological Ki-67 index determined in routine clinical setting and clinical data for 61 patients with FTC, 158 patients with FTA and 15 patients with follicular tumor of uncertain malignant potential (FT-UMP) surgically treated and diagnosed by histopathology at Karolinska University Hospital 2006-2017 (Cohort A). A previously published cohort of 109 patients with follicular tumors was re-analyzed as well (Cohort B).

**Results:**

In Cohort A, patients with FTC had a higher Ki-67 index (*p* < 0.001), larger tumor size (*p* < 0.001) and higher age at diagnosis (*p* = 0.036) compared to patients with FTA or FT-UMP. Hürthle cell differentiation, present in 50 FTA, 20 FTC and 8 FT-UMP, was associated with higher Ki-67 index (*p* = 0.009). Multivariate analysis of Cohort A identified a high Ki-67 index (odds ratio [OR]: 1.215, *p* < 0.001) and large tumor size (OR: 1.038, *p* < 0.001) as independent predictors of FTC. Results remained consistent after exclusion of Hürthle cell tumors and in pooled analysis of Cohort A + B. The area under curve of the Ki-67 index for predicting FTC was 0.722 and a cut-off for Ki-67 index at above 5% resulted in a specificity at 93% and sensitivity at 31%. Subgroup analysis of FTCs in Cohort A showed an association of higher Ki-67 index to extrathyroidal extension (*p* = 0.001) as well as widely invasive subtype (*p* = 0.019) based on the WHO 2017 classification.

**Conclusions:**

Pre-operative Ki-67 index may add diagnostic information for a subset of patients with follicular thyroid tumors.

## Introduction

The incidence of thyroid cancer has increased during the last three decades [1]. Follicular thyroid carcinoma (FTC)—the second most common type of thyroid carcinoma—accounts for approximately 10% of clinically manifest thyroid malignancies [1].

Follicular lesions of the thyroid gland also include the most common benign neoplasm—follicular thyroid adenoma (FTA) [2, 3]. Follicular tumor of uncertain malignant potential (FT-UMP), previously termed atypical follicular thyroid adenoma (AFTA), is a variant of follicular thyroid tumors with “worrisome histological features” but which lack some necessary criteria to establish a diagnosis of FTC, i.e., capsular and/or vascular invasion [2, 3]. The malignant potential for FT-UMP is regarded as low and the majority of cases have a benign course [4].

The initial diagnosis of thyroid lesions is most often done with fine needle aspiration (FNA) cytology. Its overall sensitivity is high [5] but it cannot serve to distinguish FTC from FTA [6]. Due to the ambiguous cytology of follicular lesions, a second surgical procedure with completion thyroidectomy can be required in the majority of FTC cases [5]. In the past decade, commercially available molecular testing have been developed to aid the diagnosis of thyroid nodules with indeterminate cytology [7]. Several molecular markers have been proposed to aid in the sub-classification of follicular lesions; however, data on their specific role for identifying FTC is still limited [7, 8]. In this study, we collected a large cohort of follicular thyroid tumors previously diagnosed by expert cytologists and pathologists from our institution to analyze the diagnostic value of the Ki-67 proliferation index in FNA material to differentiate FTC, alone or combined with other clinical characteristics such as age at diagnosis, gender and tumor size. In addition, we evaluated the Ki-67 index in relation to extrathyroidal extension and sub-classifications of FTC based on 2017 WHO classification system.

## Material and methods

### Patient cohort A

The Scandinavian Quality Register for Thyroid, Parathyroid and Adrenal Surgery and a local database at the Department of Pathology were used to identify all patients with follicular neoplasia surgically treated at Karolinska University Hospital during the period 2006–2017.

**Fig. 1:**
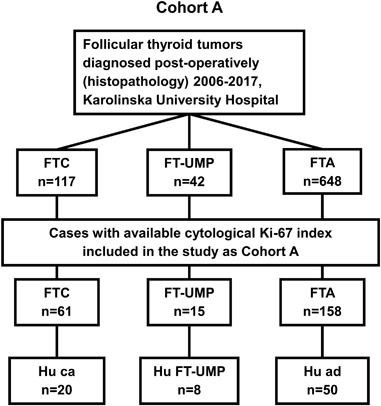
Flow chart of patient selection process in Cohort A. Inclusion of FTC and FT-UMP patients were based on the availability of Ki-67 index in cytology; a group of patients with FTA was randomly selected as control group. FTC, follicular thyroid carcinoma; FT-UMP, follicular tumor of uncertain malignant potential; FTA, follicular thyroid adenoma; Hu ca, Hürthle cell carcinoma; Hu FT-UMP, FT-UMP, Hürthle cell type; Hu ad, Hürthle cell adenoma

Inclusion criteria were a final histopathological diagnosis of FTC or FT-UMP and an available preoperative Ki-67 index given in the routine cytology report (Fig. 1). A randomly selected group of FTA was included as a control group. Exclusion criteria were missing data on Ki-67 index in cytology and concurrent thyroid malignancy other than papillary microcarcinoma; of the included cases, three patients with FTA, one patient with FT-UMP and three with FTC had concurrent papillary microcarcinoma. A total of 234 patients were included in the study following these criteria (Table 1). The study was approved by the regional ethics committee. All patients gave their informed consent for collection and analysis of clinical data prior to this study.

**Table 1:** Clinical characteristics and cytological evaluation of the 234 cases in Cohort A

	FTC	FT-UMP	FTA
Parameter	*n* = 61 (41 women)	*n* = 15 (9 women)	*n* = 158 (113 women)
Age at surgery			
Median (min.–max.)	66 years (11–92)	46 years (15–82)	54 years (16–93)
Bethesda classification			
Category I	0	0	0
Category II	1	0	3
Category III	0	4	20
Category IV	52	9	131
Category V	5	2	3
Category VI	3	0	1
Cytology			
Median Ki-67 index (min.–max.)	3% (1–30%)	2% (1–25%)	1% (0–10%)
Hürthle cell differentiation	15	5	47
Post-operative histopathology			
Hürthle cell differentiation	20	8	50
Median tumor size (min.–max.)	40 mm (17–90)	32 mm (14–60)	28 mm (7–85)
WHO 2004 classification			
Minimally invasive	19	–	–
Widely invasive	42	–	–
WHO 2017 classification			
Minimally invasive	24	–	–
Encapsulated angioinvasive	7	–	–
Widely invasive	30	–	–

*FTA* follicular thyroid adenoma, *FTC* follicular thyroid carcinoma, *FT-UMP* follicular tumor of uncertain malignant potential

Bethesda category: I = Nondiagnostic or unsatisfactory, II = Benign, III = Atypia of undetermined significance or follicular lesion of undetermined significance, IV = Follicular neoplasm or suspicious for a follicular neoplasm, V = Suspicious for malignancy, VI = Malignant

Tumors were diagnosed with routine histopathology according to the 2004 WHO classification [2]. However, included cases in the study were revisited by an experienced histopathologist for re-classification according to the 2017 WHO criteria [3]. Tumors with presence of >75% Hürthle cells were classified as Hürthle cell tumors (also referred to as oxyphilic or oncocytic tumors) [2, 3]. Follicular tumors with an uncertain relation to the capsule (extension into, but not through, the capsule) and/or worrisome features (high cellularity and Ki-67 index >5%) on histopathological evaluation were classified as FT-UMP [2, 3]. Tumor size was defined as the largest tumor diameter of the removed specimen prior to formalin fixation.

### Patient cohort B

In order to increase the statistical power and validate the results of Cohort A, re-evaluation and analysis was performed on a previously published cohort by Sofiadis et al. [9]. A total of 149 cases with follicular tumors (including Hürthle cell tumors) were re-examined with regard to histopathological evaluation based on the 2004 WHO classification [2] (Supplementary Fig. 1). Data on tumor size, age at diagnosis and gender were collected. Cases with missing data were excluded from analysis. A total of 109 cases, with 65 FTA (21 Hürthle cell adenoma), 24 FTC (10 Hürthle cell carcinoma) and 20 AFTA (7 AFTA with Hürthle cell type), were included for separate univariate analysis and pooled multivariate analyses of Cohort A + B.

### Cytology and Ki-67 immunocytochemistry

FNA cytology was performed and evaluated as part of the routine clinical workup. The aspirated material was mainly used for cytomorphological evaluation. In a subgroup of patients, a part of the aspirate was used to determine Ki-67 proliferation index by immunocytochemistry. In short, air-dried smears were fixed in buffered 4% formaldehyde solution followed by methanol and acetone. The monoclonal Ki-67 antibody (clone MIB-1, DAKO M7240) was used with a dilution of 1:200. In Cohort A, prior to 2010, the smears were manually stained with immunoperoxidase-avidin-biotin technique. Since 2010 the staining has been performed by an automated BOND-MAX stainer (Leica Biosystem, Germany) with standardized methodology with BOND polymer refine detection kit, poly-HRP (horse-radish-peroxidase) reagent and diaminobezidin (DAB). Scoring was performed by calculating the percentage of positive cells (brown stained nuclei) by counting at least 200 tumor cells. Analyses of reactive lymph nodes were included as positive control which revealed distinct nuclear staining and omission of the primary antibody served as negative control. In Cohort B the immunological technique was performed as previously described by Sofiadis et al. [9].

### Ki-67 immunohistochemistry

For a total of 138 cases in Cohort A, Ki-67 immunohistochemistry was performed on formalin-fixed paraffin-embedded histopathological specimens. The immunohistochemistry was performed using an accredited methodology used in clinical routine practice with CONFIRM anti-Ki-67 (30-9) Rabbit Monoclonal Primary Antibody and stained with Ventana automated slide stainer (Ventana Medical Systems, Inc., USA). Analyses of lymph node and tonsils were included as positive controls and omission of the primary antibody served as negative control. Scoring was performed by calculating the percentage of positive cells (brown stained nuclei) in hotspot areas in at least 2000 cells.

### Statistical analyses

All statistical analyses were performed using IBM SPSS Statistics version 24.0 (IBM, Armonk, NY, USA). Univariate analyses were performed with Mann–Whitney *U*-test, Kruskal-Wallis test and Chi-square test. Correlation was analyzed with Spearman’s rank correlation coefficient. A binary logistic regression model was used for multivariate analyses. Receiver operating characteristic (ROC) curves and area under curve (AUC) were used to assess diagnostic value of Ki-67 index and sensitivity, specificity, positive predictive value (PPV), negative predictive value (NPV) and accuracy were calculated.

## Results

### Patient cohort A

The clinical characteristics of Cohort A are summarized in Table 1. Based on FNA cytology, 216/234 cases (92%) were classified as Bethesda category IV (follicular neoplasm/suspicious for a follicular neoplasm) or category III (atypia or follicular lesion of undetermined significance). Of the carcinomas, 85% were classified as Bethesda IV. The distribution of cytological Ki-67 index across the different Bethesda categories are detailed in Supplementary Table 1.

**Fig. 2:**
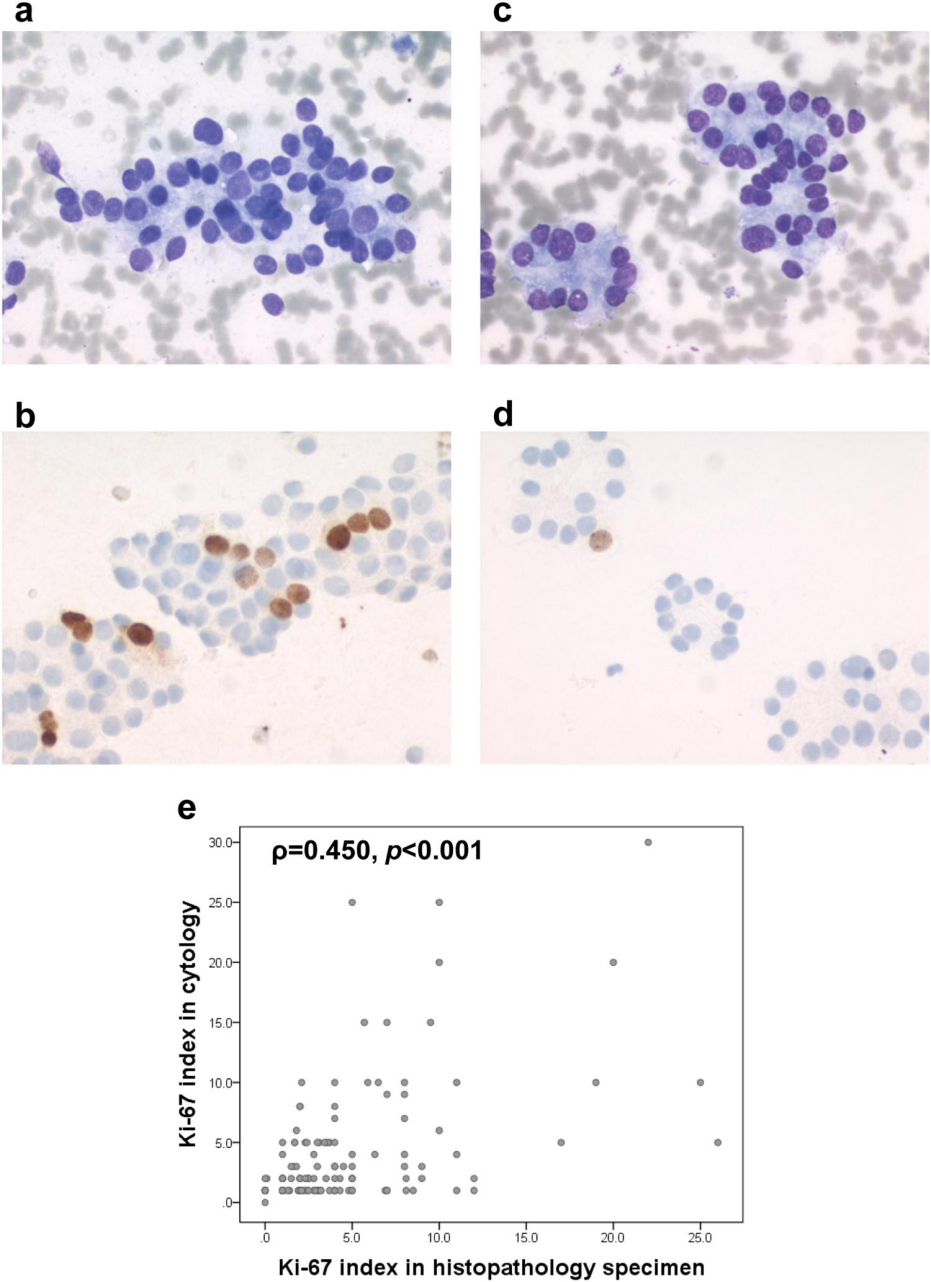
FNA smears from a non-Hürthle cell FTC (**a**, **b**) with high Ki-67 index of 10% (**b**) and a non-Hürthle cell FTA (**c**, **d**) with low Ki-67 index at 1% (**d**). Cytomorphology based on May-Grünwald/Giemsa staining (**a**, **c**) and assessment of Ki-67 proliferation index by MIB-1 immuncytochemistry (**b**, **d**) are shown at x20 magnification. **e** Comparison of Ki-67 index determined by immunocytochemistry in pre-operative cytology specimens and by immunohistochemistry in post-operative specimens from 138 cases in Cohort A. FTC, follicular thyroid carcinoma; FTA, follicular thyroid adenoma

**Fig. 3:**
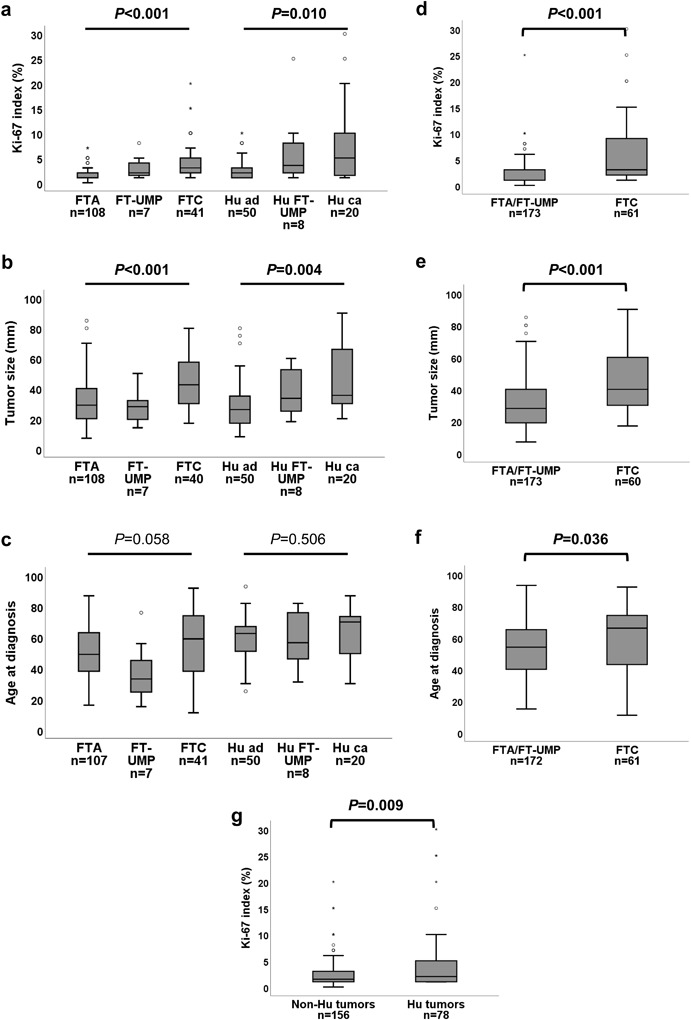
Box-plots illustrating the distribution of Ki-67 index in cytology, tumor size and age at diagnosis across the diagnostic groups of follicular thyroid tumors subgrouped according to Hürthle cell differentiation in Cohort A (**a**–**c**). The distribution of Ki-67 index, tumor size and age at diagnosis of FTC and FTA/FT-UMP in the whole Cohort A (**d**–**f**), and the distribution of Ki-67 index in Hürthle cell and non-Hürthle cell tumors (**g**). Bars indicate non-outlier range and boxes indicate interquartile. Medians are illustrated as horizontal lines within boxes. Bullets indicate outliers. FTC, follicular thyroid carcinoma; FT-UMP, follicular tumor of uncertain malignant potential; FTA, follicular thyroid adenoma; Hu ca, Hürthle cell carcinoma; Hu FT-UMP, FT-UMP, Hürthle cell type; Hu ad, Hürthle cell adenoma; Hu tumors, Hürthle cell tumors

Examples of Ki-67 immunocytochemistry using the MIB-1 antibody in FNA smears are presented in Fig. 2a–d together with corresponding cytomorphology. The cytological Ki-67 index and tumor size were found to be significantly higher in carcinomas with or without Hürthle cell differentiation (Fig. 3a, b, Supplementary Table 1), while no difference was observed for the distribution of age at diagnosis (Fig. 3c). The carcinomas exhibited independent of Hürthle cell differentiation, higher cytological Ki-67 index, larger tumor sizes and higher age at diagnosis than FTA and FT-UMP (Fig. 3d–f). In addition, Hürthle cell differentiated tumors generally had a higher median Ki-67 index than non-Hürthle cell tumors (Fig. 3g). The distribution of gender did not differ between groups (*p* = 0.629).

Ki-67 index in FNA smears and tumor size were identified as independent predictors of FTC (including Hürthle cell carcinoma) in a multivariable model with Ki-67 index, tumor size, age and gender. Results remained consistent when omitting the Hürthle cell tumors from the analysis (Table 2).

**Table 2:** Multivariate analyses with binary logistic regression for prediction of FTC in Cohort A, before and after exclusion of Hürthle cell tumors

Analysis variables	*B*	SEM	exp B (OR)	CI 95%	*p*-value
FTA/FT-UMP vs. FTC (*n* = 232^a^)					
Ki-67 index in cytology	0.195	0.049	1.215	1.102–1.338	**<0.001**
Tumor size	0.037	0.010	1.038	1.018–1.059	**<0.001**
Age at surgery	0.001	0.009	1.001	0.983–1.019	0.905
Gender	0.050	0.366	1.051	0.513–2.155	0.892
Non-Hürthle cell FTA/FT-UMP vs. FTC (*n* = 154^a^)					
Ki-67 index in cytology	0.431	0.111	1.539	1.238–1.913	**<0.001**
Tumor size	0.041	0.013	1.042	1.015–1.070	**0.002**
Age at surgery	−0.001	0.011	0.999	0.978–1.021	0.930
Gender	0.079	0.470	1.082	0.431–2.718	0.867

*FTA* follicular thyroid adenoma, *FTC* follicular thyroid carcinoma, *FT-UMP* follicular tumor of uncertain malignant potential, *B* unstandardized Beta, *SEM* standard error of the mean, *exp B (OR)* odds ratio, *CI 95%* 95% confidence interval

^a^Two cases without complete data

Bold values indicate significant variables

When evaluating the predictive value of the Ki-67 index for FTC (including Hürthle cell carcinoma) with ROC-analysis, the area under curve (AUC) was 0.722 for the whole Cohort A (Supplementary Figure 2). Sensitivities, specificities, PPV, NPV, and accuracy were subsequently calculated at cut-offs of Ki-67 index set at above 4 and 5% for the whole Cohort A and with stratification based on Bethesda categories III and IV (Table 3). With the cut-off set at above 5%, the specificity increased (93%) while the sensitivity decreased (31%), accuracy was 77%. The diagnostic values were similar when stratifying for Bethesda categories III or IV (*n* = 216) and Bethesda IV only (*n* = 192). Also, using the same cut-off at above 5%, only 8/158 patients with FTA (including 6 Hürthle cell adenoma) had a Ki-67 index above 5%, while 19/61 patients with FTC (including 9 Hürthle cell carcinoma) exhibited a Ki-67 index higher than 5%.

**Table 3:** Sensitivities, specificities, positive (PPV), negative predictive values (NPV) and accuracy for predicting FTC based on Ki-67 index cut-off at above 4 and 5% in Cohort A

	*n*	Sensitivity (%)	Specificity (%)	PPV (%)	NPV (%)	Accuracy (%)
All follicular tumors	234					
Above 4%		43	87	53	81	75
Above 5%		31	93	61	79	77
Bethesda category III or IV	216					
Above 4%		38	87	49	82	75
Above 5%		25	93	57	80	77
Bethesda category IV	192					
Above 4%		38	86	50	79	73
Above 5%		25	93	57	77	74

Bethesda Category III = Atypia of undetermined significance or follicular lesion of undetermined significance

Bethesda Category IV = Follicular neoplasm or suspicious for a follicular neoplasm

*FTC* follicular thyroid carcinoma, *n* = number of cases

The Ki-67 index determined in cytology specimens correlated significantly with the Ki-67 index from immunohistochemical analyses of post-operative specimens for the 138 cases where both were assessed (Spearman’s rank correlation coefficient *ρ* = 0.450, *p* < 0.001; Fig. 2e).

**Fig. 4:**
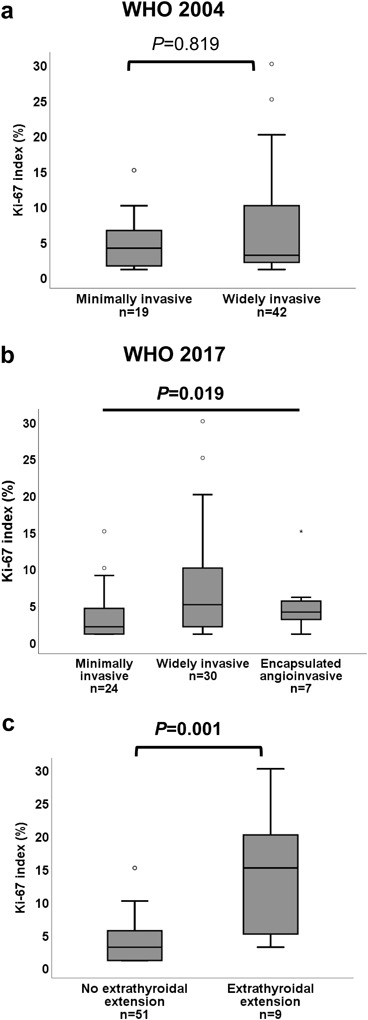
Box-plots illustrating the distribution of Ki-67 index in the subgroups of FTC according to WHO 2004 (**a**) and WHO 2017 (**b**). A significant difference in Ki-67 index was only observed between the subgroups according to WHO 2017. The distribution of Ki-67 index in FTCs with and without extrathyroidal extension (**c**). Bars indicate non-outlier range and boxes indicate interquartile. Medians are illustrated as horizontal lines within boxes. Bullets indicate outliers. FTC, follicular thyroid carcinoma

In subgroup analyses of FTCs (including Hürthle cell carcinoma) in Cohort A, no significant difference in Ki-67 index between minimally (median: 4%; min–max: 1–15%) and widely invasive (median: 3%; min–max: 1–30%) FTC was observed based on the WHO 2004 classification (Fig. 4a). However, after re-classification according to WHO 2017, a difference in Ki-67 index was observed between the subgroups of FTC (*p* = 0.019), with post-hoc test revealing a statistically significant difference between minimally (median: 2%; min–max: 1–15%) and widely invasive (median: 5%; min–max: 1–30%) FTC (*p* = 0.016, Fig. 4b). In addition, the presence of extrathyroidal extension was found to be associated with a higher Ki-67 index (Fig. 4c).

### Patient Cohort B

Subsequent analysis of Cohort B (*n* = 109) verified the results from Cohort A. The FTC group had a significantly higher median Ki-67 index (*p* = 0.003), tumor size (*p* = 0.010) and age at diagnosis (*p* = 0.011) as compared to FTA or AFTA when pooling Hürthle cell tumors together with non- Hürthle cell tumors (Supplementary Figure 3). No difference in Ki-67 index was observed between Hürthle cell tumors and non-Hürthle cell tumors in Cohort B (*p* = 0.729). There was also a significant difference in gender distribution between the two groups (6% males in FTA/AFTA group versus 38% males in FTC group, *p* < 0.001), which was not observed in Cohort A. Pooled multivariate analysis of Cohort A + B showed consistent results with analyses of Cohort A only, with Ki-67-index and tumor size identified as independent predictors of FTC (Supplementary Table 2).

## Discussion

In this study we found that the Ki-67 index in FNA material has a predictive value in the diagnosis of FTC. Multivariate analysis identified the Ki-67 index and tumor size as independent predictors of FTC in Cohort A and results remained consistent after inclusion of Cohort B in the multivariate analysis. In addition, we found that higher Ki-67 index was associated to widely invasive subtype based on 2017 WHO classification system as well as extrathyroidal extension in FTC.

Pre-operative diagnosis of follicular neoplasia is a challenge. The goal of an optimal diagnostic test is to achieve best management and avoid overtreatment [8]. The criteria distinguishing FTC from FTA and FT-UMP are based on histopathological findings such as vascular and/or capsular invasion [2, 3]. Consequently, the majority of patients with FTC will need to undergo two surgical procedures [5]. A number of studies have been conducted on the Ki-67 proliferation index in follicular thyroid tumors using post-operative specimens [10–17] but just a few on Ki-67 in pre-operative settings. Some have reported that Ki-67 is potentially useful in aiding the diagnosis of follicular thyroid tumors [18–21], but the results are inconsistent [9, 22]. Based on the ROC-analysis performed on Cohort A, we found that the Ki-67 index was fair as a diagnostic test with AUC at 0.722. A high Ki-67 index contributed to the stratification of malignancy risk. With a cut-off set at above 5%, the specificity and sensitivity for the cases with Bethesda categories III/IV in Cohort A was 93 and 25%, respectively while the accuracy was 77%. One in four in our FTC cohort could thus be identified with this analysis, without reaching a high rate of false positives. Since the prevalence of FTA is approximately 6–7 times higher than that of FTC at our institution, a high specificity is important to avoid overdiagnosis. The overlap of low level Ki-67 index between FTA/FT-UMP and FTC was however significant and outliers were present in both groups, in line with reports by others [22]. Although previous studies have shown a higher accuracy of Ki-67 at 0.804 with sensitivity at 61% and specificity at 75% [23], it should be noted that the studies included are predominately based on papillary thyroid carcinoma and the Ki-67 index based on immunohistochemistry [23, 24].

In this study we observed a correlation between Ki-67 index determined in FNA material and postoperative specimens, respectively (Fig. 2e). However, the two types of analyses are not fully comparable in that the cytological Ki-67 index is calculated as a mean for the analyzed cells in the smear, while the post-operative assessment of Ki-67 index is analyzed in “hot spot” areas of the section. Also, the cytological scoring is based on counting 200 cells, while post-operative scoring is based on 2000 cells.

Our results also suggest, consistent with earlier findings, that the predictive value of Ki-67 index has to be assessed differently for Hürthle cell and non-Hürthle cell tumors [16, 20]. The Hürthle cell tumors had an overall higher Ki-67 index compared to non-Hürthle cell tumors in Cohort A and exclusion of these tumors from the analysis slightly increased the predictive value of a high Ki-67 index in multivariate analysis. However, the number of Hürthle cell tumors in this cohort is too limited to provide any further information about the value of the Ki-67 index in distinguishing Hürthle cell carcinoma from Hürthle cell adenoma and FT-UMP with Hürthle cell type.

Interestingly, pre-operative Ki-67 index was significantly higher in widely invasive FTCs than minimally invasive FTCs when stratifying the tumors based on the novel 2017 WHO criteria, but no difference was seen when applying the 2004 WHO criteria. This could be due to the more stringent 2017 classification of widely invasive FTCs, in which tumors must display both angio-invasion as well as capsular invasion, and not just multiple foci with one of the above mentioned criteria. When we analyzed prognostic factors in minimally invasive FTC defined by the 2004 WHO criteria in an earlier study, combined capsular and vascular invasion was identified as a negative prognostic factor [25].

Along this observation, we also found that the presence of extrathyroidal extension was associated to a higher Ki-67 index in our cohort of FTC. Although previous studies are mainly conducted on papillary thyroid carcinomas and Ki-67 index based on immunohistochemistry, the results are in line with previous reports of the association of Ki-67 to prognostic features, including extrathyroidal extension [23, 26, 27].

Of the commercially available molecular tests that have been developed and validated, the next-generation sequencing-based multi-genetic panel ThyroSeq seems to have the most promising results [7, 28, 29]. Results have shown a sensitivity of up to 90% and specificity up to 93% for Bethesda Category III and IV [28, 30]. However, available data are still limited for the cases with FTC since most validated cases of thyroid malignancies were represented by papillary thyroid carcinoma [7, 28, 30].

The strength of the present study is the relatively large patient cohort and the high concordance rate of cytological identification of follicular neoplasia. In our Cohort A, a total of 82% were diagnosed as Bethesda Category IV in cytology and only 1/61 patients with a final diagnosis of FTC had a Bethesda Category of II (benign). Another strength of the study is the consistent finding of the predictive value of Ki-67 staining gained by re-evaluation of the previously published Cohort B of follicular thyroid neoplasms, supporting the reliability of the results. It should be noted however, that Sofiadis et al. did not reach statistical significance in the original published work [9], which could be the attributed to the differences in study design and the subgrouping of Hürthle cell tumors. However, scrutinizing the data shows a similar trend for higher Ki-67 index in FTC. In our Cohort A, we observed that even when subgrouping after Hürthle cell differentiation, the carcinomas exhibited a higher Ki-67 index.

The main limitation of this study is the fact that cytological Ki-67 index was not determined in every patient with indeterminate cytology (Bethesda Category III and IV). Another possible limitation is the likelihood of discordance in distribution of Bethesda categories between institutions. Although Ki-67 is widely used as a diagnostic and prognostic marker in different types of malignancy [31], its predictive value may differ between institutions due to differences in patient volumes and the distribution across different Bethesda categories [8]. Moreover, from a pre-operative perspective, an eventual coupling between Ki-67 index and prognostic ultrasonographical parameters could not be assessed due to the limited number of cases in our cohort investigated in a standardized ultrasonographical manner.

To conclude, we identified a high Ki-67 index in FNA smears and tumor size as predictors of FTC in follicular thyroid tumors. In FTC, we also found a high Ki-67 index to be associated with extrathyroidal extension as well as widely invasive subtype based on the 2017 WHO classification. Analysis of the Ki-67 index has a relatively low cost and may contribute to the subtyping of follicular thyroid tumors for a subset of patients. However, our results need to be validated prospectively and other preoperative biomarkers for FTC are needed to further increase sensitivity.

## Electronic supplementary material

Supplementary Fig. 1

Supplementary Fig. 2

Supplementary Fig. 3

Supplementary Table 1

Supplementary Table 2
