# Supplementary material for: High Ki-67 index in fine needle aspiration cytology of follicular thyroid tumors is associated with increased risk of carcinoma
Source: Endocrine. 2018 May 23;61(2):293–302. doi: 10.1007/s12020-018-1627-z (PMC6061212; doi:10.1007/s12020-018-1627-z)
Supplement: Supplementary file 1 — Supplementary Fig. 1 [file 12020_2018_1627_MOESM1_ESM.pdf]

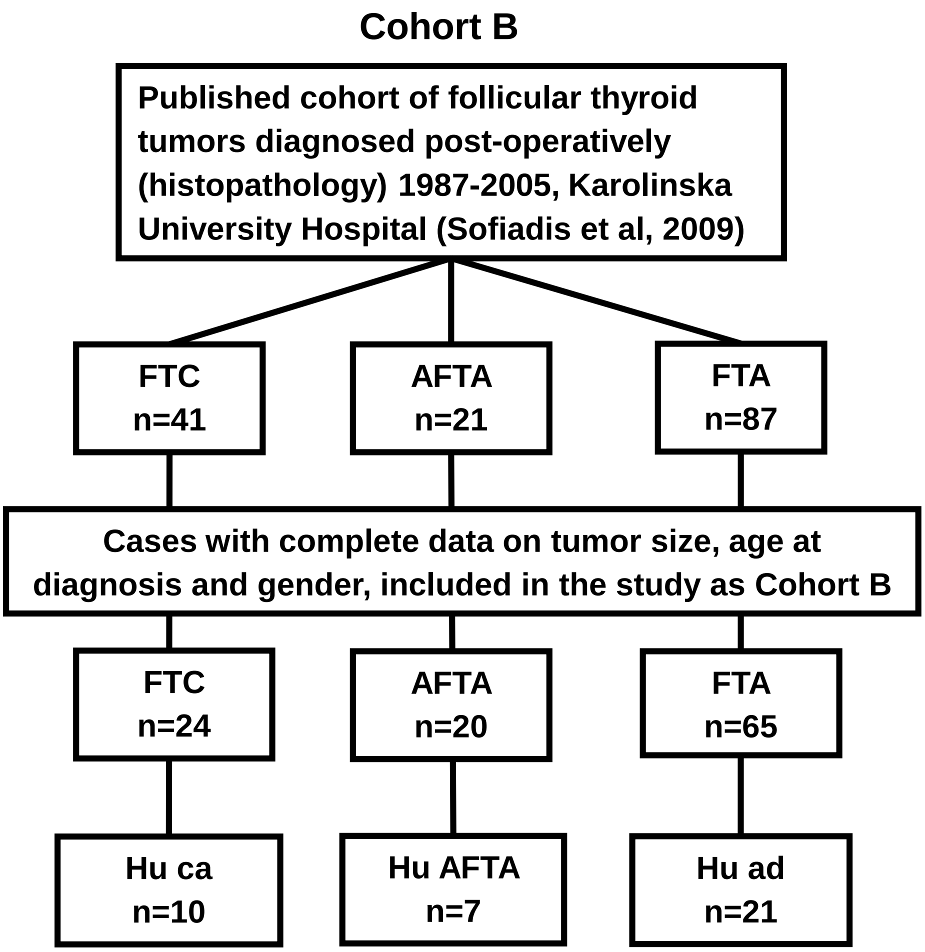

Supplementary Figure 1. A previously published cohort of follicular thyroid tumors (including Hürthle cell tumors) from Karolinska University Hospital was re-evaluated and re-analyzed as Cohort B (Sofiadis et al, Int J Oncol. 2009 [9]). FTC=follicular thyroid carcinoma; AFTA=atypical follicular thyroid adenoma; FTA=follicular thyroid adenoma; Hu ca=Hürthle cell carcinoma; Hu AFTA=AFTA of Hürthle cell type; Hu ad= Hürthle cell adenoma
