# Supplementary material for: High Ki-67 index in fine needle aspiration cytology of follicular thyroid tumors is associated with increased risk of carcinoma
Source: Endocrine. 2018 May 23;61(2):293–302. doi: 10.1007/s12020-018-1627-z (PMC6061212; doi:10.1007/s12020-018-1627-z)
Supplement: Supplementary file 2 — Supplementary Fig. 2 [file 12020_2018_1627_MOESM2_ESM.pdf]

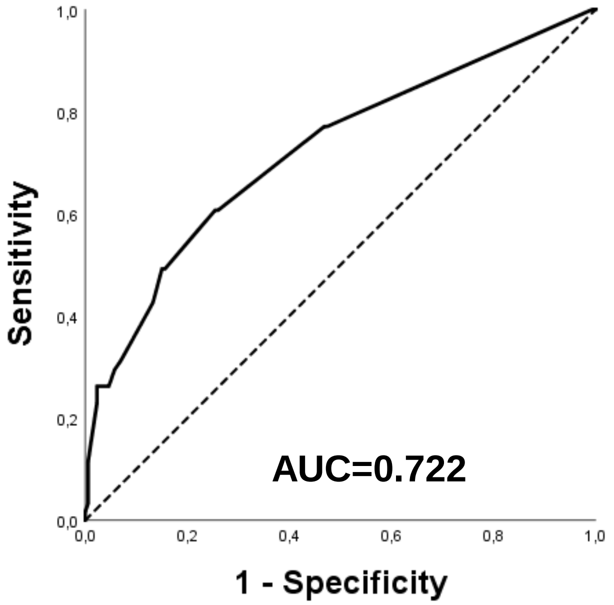

Supplementary Figure 2. Evaluation of the predictive value of cytological Ki-67 index for follicular thyroid carcinoma (FTC) by ROC analysis. Area under curve (AUC) was 0.722 in analysis of all follicular tumors in Cohort A (n=234). Broken line indicates reference line for AUC at 0.5.
