# Supplementary material for: High Ki-67 index in fine needle aspiration cytology of follicular thyroid tumors is associated with increased risk of carcinoma
Source: Endocrine. 2018 May 23;61(2):293–302. doi: 10.1007/s12020-018-1627-z (PMC6061212; doi:10.1007/s12020-018-1627-z)
Supplement: Supplementary file 3 — Supplementary Fig. 3 [file 12020_2018_1627_MOESM3_ESM.pdf]

Cohort B

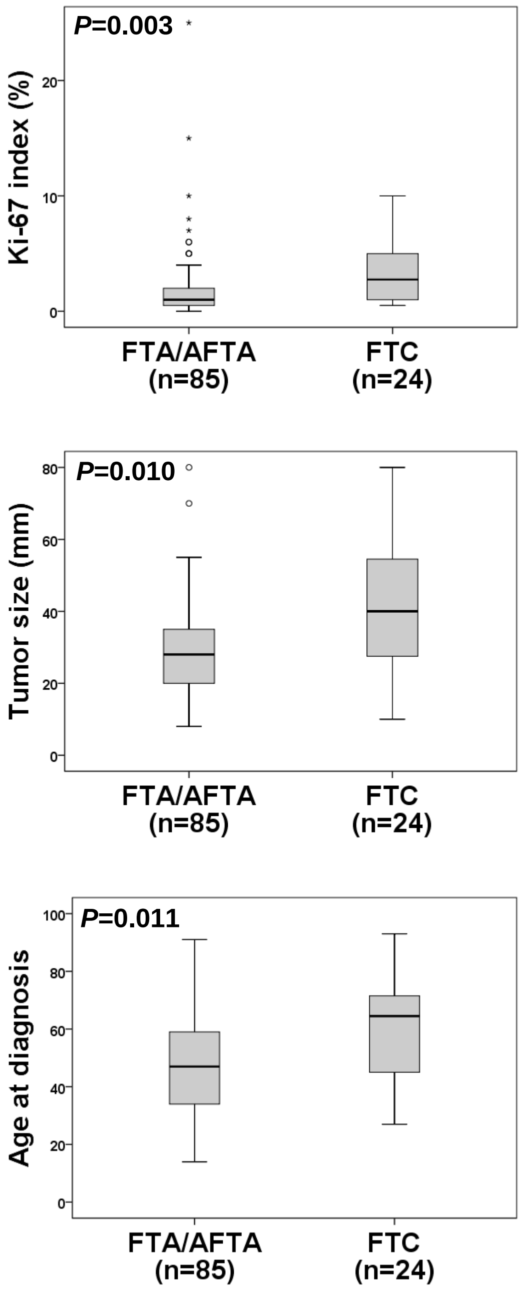

Supplementary Figure 3. Box-plots illustrating the distribution of cytological Ki-67 index, tumor size and age at diagnosis in FTA/AFTA and FTC in Cohort B. Bars indicate non-outlier range and boxes indicate interquartile. Medians are illustrated as horizontal lines within boxes. Bullets indicate outliers. FTC=follicular thyroid carcinoma; AFTA=atypical follicular thyroid adenoma; FTA=follicular thyroid adenoma
